# Selected Tribological Properties and Vibrations in the Base Resonance Zone of the Polymer Composite Used in the Aviation Industry

**DOI:** 10.3390/ma13061364

**Published:** 2020-03-18

**Authors:** Aneta Krzyzak, Ewelina Kosicka, Marek Borowiec, Robert Szczepaniak

**Affiliations:** 1 Department of Airframe and Engine, Military University of Aviation, 80-521 Dęblin, Poland; r.szczepaniak@law.mil.pl; 2 Department of Production Engineering, Lublin University of Technology, 20-618 Lublin, Poland; e.kosicka@pollub.pl; 3 Department of Applied Mechanics, Lublin University of Technology, 20-618 Lublin, Poland; m.borowiec@pollub.pl

**Keywords:** composite material, tribology, vibrations, resonance zone

## Abstract

The revolution in the global market of composite materials is evidenced by their increasing use in such segments as the transport, aviation, and wind industries. The innovative aspect of this research is the methodology approach, based on the simultaneous analysis of mechanical and tribological loads of composite materials, which are intended for practical use in the construction of aviation parts. Simultaneously, the methodology allows the composition of the composites used in aviation to be optimized. Therefore, the presented tests show the undefined properties of the new material, which are necessary for verification at the application stage. They are also a starting point for further research planned by the authors related to the improvement of the tribological properties of this material. In this article, the selected mechanical and tribological properties of an aviation polymer composite are investigated with the matrix of L285-cured hardener H286 and six reinforcement layers of carbon fabric GG 280P/T. The structure of a polymer composite has a significant influence on its mechanical properties; thus, a tribological analysis in the context of abrasive wear in reciprocating the movement for the specified polymer composite was performed. Moreover, the research was expanded to dynamic analysis for the discussed composite. This is crucial knowledge of material dynamics in the context of aviation design for the conditions of resonance vibrations. For this reason, experimental dynamical investigations were performed to determine the basic resonance of the material and its dynamics behavior response. The research confirmed the assumed hypotheses related to the abrasive wear process for the newly developed material, as well as reporting an empirical evaluation of the dependencies of the resonance zone from the fabric orientation sets.

## 1. Introduction

The multifaceted approach to conducting improvements in various industry sectors is manifested both in organizational aspects, by forcing the improvement of the quality of implemented tasks (an example may be striving to eliminate the occurrence of machinery or equipment failures [1]), as well as in design aspects [2], which includes the optimization of previously developed structures [3] and technology [4] and carrying out modifications of the materials used [5,6]. Activities related to material improvements determine the dynamics of material engineering development, while assumptions related to the operating conditions of the components made of a given material determine the direction of the changes and expectations of its final properties. These are determined after a wide range of tests (often standardized) are performed, which indicate the dependency between the preparation method of the material and its characteristics, often taking into account external factors [7] such as UV radiation [8] and humidity [9].

The role of material testing increases particularly in the case of high reliability requirements for components made of this material. Among the various industrial sectors, the aviation industry is one that requires absolute reliability. For this reason, all potential solutions used in this industry, including materials, must be thoroughly tested, which indicates the topicality of the topic taken in this article. Additionally, dynamics analysis is crucial from a system stability point of view. It is impossible to exclude complex excitations of the structure from external sources; hence, the material can manifest the existence of various nonlinearities. Moreover, the structure may behave noisily or chaotically [9] and can respond by non-trivial dependence of amplitude and frequency, or even by the occurrence of multiple solutions [10,11].

According to the hypothesis made at the planning stage of the research part for this paper on the new composite material, the authors expected the following increase in abrasive wear and the maximum roughness depth R_max_ as the number of cycles increases, as well as the observation of resonance zone movements while the structure vibrates, due to fabric orientation sets. In this article, the preliminary results are described. The research aim was to develop a new composite material characterized by high tribological resistance and required mechanical properties associated with resonance vibrations. The resin used can be used to manufacture components for gliders, powered sailplanes, and motor-powered aeroplanes. The authors did not find other publications on tribological research in connection with resonance studies of parts made of this resin, hardener, or fabric in specific configurations. The obtained results are the first stage of planned research consisting of carrying out modifications to improve the properties of the aviation composite.

### 1.1. Polymer Composites Used in Aviation

By using various materials for warp or reinforcement, there are unlimited possibilities to modify the properties of composites. This influences the dynamics of the increase in interest in these materials observed in global industry trends. Research carried out in the field of developing high-strength and highly modular constructions, while reducing the specific gravity of composites, opens up the perspective of increasingly bolder use of them in the construction, automotive, and aerospace industries [11].

The main motivation toward the development of new materials for the aviation industry is the reduction of aircraft weight, extending the time of reliable operation of the parts, improving fuel efficiency, and thus reducing aircraft operating costs [12,13,14]. These materials can be found in both internal and external aircraft constructions; in internal structures (secondary structures), they include cabins, floorboards, or armchairs, but it is the external (primary) structures that constitute the main market for composite aviation materials.

Primary examples of the implementation of composite materials in the structure of aircraft are the Airbus A380 and the Boeing 787 Dreamliner. In the former, the world’s largest passenger aircraft, the composites constitute 25% of the weight of the entire aircraft, where as much as 22% are fibrous composites with an epoxy resin matrix and the reinforcement is Carbon Fiber-Reinforced Polymer (CFRP) carbon fibers. The carbon fibers used ensure high rigidity (935 GPa), and their density is 60% of the density of aluminum (achieving a 40% lighter weight than is the case with constructions made of aluminum). An additional argument in favor of the use of CFRP composites is the relative ease of obtaining the complex shapes of the components. In addition, they are characterized by good thermal and electrical conductivity and X-ray absorption, as well as the ability to dampen vibrations [15].

**Figure 1:**
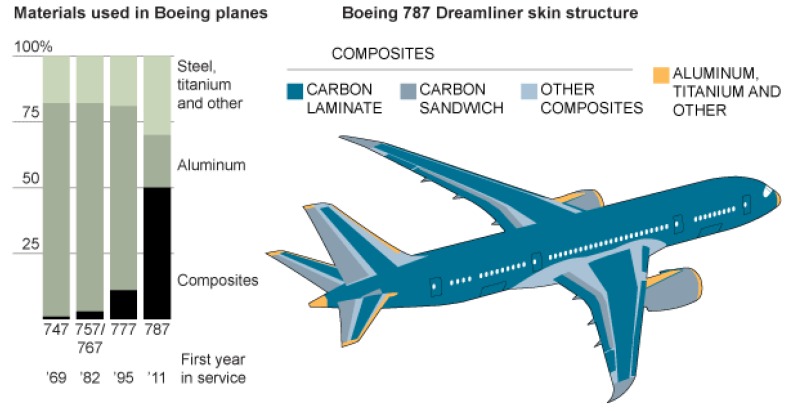
Materials used in the Boeing 787 Dreamliner skin structure [19].

The second passenger aircraft, which can boast of functioning implementations of composite components, is the Boeing 787 Dreamliner (Figure 1), which has been designated as a ground-breaking application of composite materials, inspiring the use of these materials deep in the currents of commercial products [16]. This aircraft accounts for up to 50% by mass (and 80% by volume) of the composite materials applied to the main airframe structures [17]. As emphasized by Merkisz and Bajerlein in [18], this results, among other things, in a significant reduction in aircraft weight, high corrosion resistance, easier production of complex shapes, and a reduction in assembly time compared to earlier conventional constructions. However, the estimated fuel consumption during operation is about 20% lower compared to the same class of aircraft.

**Figure 2:**
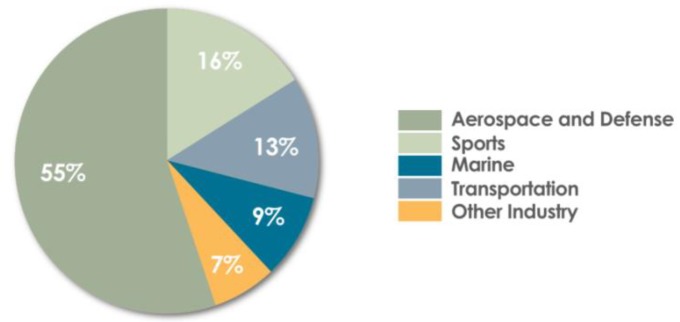
Areas of application for carbon fibers (based on: [22]).

The conducted literature analysis has allowed us to observe that, in aviation polymer composites, carbon fibers are often used as reinforcement. They are obtained mainly as a result of polyacrylonitrile pyrolysis, and their properties are shaped by the production parameters used [20]. They are characterized by good heat and chemical resistance, as evidenced by the lack of changes in the non-oxidizing atmosphere up to 2000 °C, unlike glass or aramid fibers [21]. In addition, they are characterized by low density, good thermal and electrical conductivity, and when used as friction materials, they show a low coefficient of friction. In addition, these fibers have the ability to damp vibrations and lower X-ray absorption. For this reason, their widespread use in the production of composite materials in global terms can be observed, as shown in Figure 2.

Carbon fabrics, being reinforcements made on the basis of carbon fibers, have been used in aviation solutions, and it is appropriate to mention where their weaves have been used. The discussed weaves are as follows:

—Canvas (PLAIN); 

—Oblique (TWILL);

—Satin (SATIN).

These weaves also have different weights and thicknesses.

It is also worth mentioning that the specifications in the context of the fabric used for the composite material produced should include the number of layers used or the way the subsequent reinforcement layers are laid. It is obvious the composite preparation process varies and depends on the materials used, as well as the recommendation results from the intended application of a composite.

### 1.2. Tribological Properties

Friction is considered one of the most common phenomena occurring in nature, and there has been an intensification of research in this area aimed at eliminating, or at least reducing, its formation. The problems of the occurrence of tribological wear of machine and device components ultimately translate into performance, as well as operational, reliability. The occurrence of many machine motion units necessitates limiting the formation of this irreversible phenomenon, which is achieved, for example, by using construction materials with improved anti-wear properties [23]. Tribological research in industrial conditions is difficult and expensive; therefore, the analysis of friction and wear processes is carried out on designed friction devices (also called tribometers or tribotesters). Their task is to map the association of friction through model experiments, which is why they have different designs and modes of action.

The up-to-date analysis of issues covering tribological aspects (e.g., [24,25,26]) is evidenced by publications of various scientific centers, in which the results of materials engineering research are analyzed, including considerations on how to improve the tribological properties of new materials [27,28] while determining the impact on their mechanical properties. The observed intensification in the development of material engineering in aviation, apart from defining the obtained mechanical properties for a given material, also requires the determination of tribological properties, due to the further possibilities of using these materials for structural solutions in which friction occurs.

The resin used is approved by the German Federal Aviation Office and it meets the standards that allow it to be used to make parts for gliders, powered sailplanes, and motor-powered airplanes. However, no publications can be found regarding the abrasive wear of composites made of this resin.

## 2. Measurements Methodology

### 2.1. Material Tested

Polymer composites were used to conduct this research. For the preparation of composites by hand lamination, an epoxy resin with the trade name L285, together with the hardener H285, were used, as well as carbon fabric GG 280 P/T. Due to the target use of composites for the production of aircraft components, the carbon fabric GG 280 P/T (twill 2/2, fiber 3K 200 [tex], 220 [g/m^2^]) used to make the composites had the appropriate certificates. The air bubbles formed in the matrix were removed prior to curing using ultrasonic waves. The fabric was arranged in the configuration: 0/22.5/45/0/22.5/45. Such fiber configurations, as well as the type of resin fabric, were determined by laminates applied in the aviation industry, especially in helicopter PZL SW-4. The composite material was manufactured by combining the laminating method, preparing the consecutive fabric layers impregnated by resin, and finally, pressing the structure by means of the hydraulic press PDM–50S Mecamaq at a pressure level of 2.5 MPa. The polymer composite was not affected by environmental conditions; it was not exposed to external factors such as changes in temperature, humidity or UV radiation. Due to the lack of standards specifying the dimensions of the samples used for the planned tests, the samples’ dimensions were assumed for both tests, the tribological 125 × 25 × 2.5 mm and the dynamical (see Table 1), where the frequency of vibrations in the first resonance zone was investigated. The assumed dimensions were obtained in the process of cutting with an abrasive water jet. The research was carried out in the Department of Airframe and Engine (Military University of Aviation, Dęblin), the Department of Applied Mechanics (Lublin University of Technology, Lublin), and the Department of Production Engineering (Lublin University of Technology, Lublin).

### 2.2. The Test Stand for Tribological Tests

**Figure 3:**
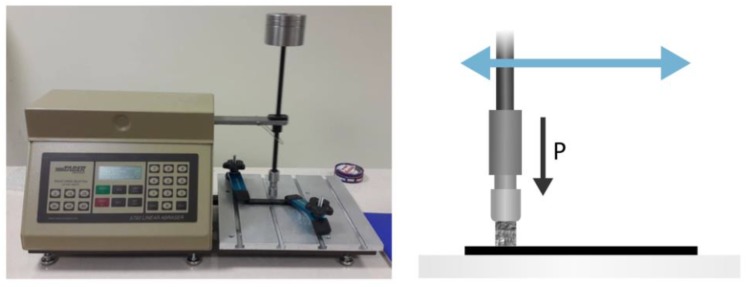
The Taber Linear Abraser model 5750.

The produced samples were tested in conditions of abrasive wear in reciprocating motion using a tribotester Taber Linear Abraser model 5750 (Figure 3). The abrasive stone used in the test bench had a diameter of 6.6 mm and a gradation of 200. The friction path was 101.6 mm/cycle, and the total load was 1850 g. After 100, 300, 600, 1000, and 1500 cycles, the mass of the samples was determined using a precision weight laboratory. After each measurement, the friction products were removed and, according to the manufacturer’s instructions, the abrasive stone and the sample were cleaned, and then the selected parameters of the wear path roughness, which were taken by the counter sample, were measured using the MicroProf 100 FRT optical profilometer. The abrasive wear of the composite was determined as a weight loss. Basic roughness parameters were determined, such as the arithmetical mean height (*R_a_*), the maximum height of the profile (*R_z_*), and maximum roughness depth (R_max_). The R_max_ parameter is understood as the largest recess in the material after the friction process. Surface parameters the arithmetical mean height (*R_a_*) and the maximum height of the profile (*R_z_*) were made in accordance with the norm PN-EN ISO 4287, using the following relationship [29]:

(1) $$R_{a}=\frac{1}{l}\int_{0}^{l}\left|Z\left(x\right)\right|dx,$$

(2) $$R_{z}=R_{p}+R_{d},$$

where: *l* is the length of the elementary segment (m); *Z* is the height of profile elements (m); *R_p_* is the maximum profile peak height (m); *R_d_* is the maximum profile valley depth (m).

### 2.3. The Resonance Frequency Estimation by the Analytical Approach

Each body or body system has its own natural frequency. Its value is influenced by both the body shape and the physical properties of the system, resulting, for example, from the material used. Knowledge of the natural frequencies allows avoiding the undesirable resonance phenomenon in the construction of machines and devices, which can lead to their damage. For this reason, the role of natural vibrations in the area of aviation is raised primarily in the context of safety aspects.

In [30], the impact of the emergency condition of the aircraft structure on the change of its natural vibrations was mentioned. As the author emphasizes, this is especially important in the case of military aircraft used for combat tasks, when there is a risk of local violations of the structure.

On the other hand, the authors of [31] refer to the necessity of conducting vibration tests before issuing the type for the aircraft being put into service. They emphasize that the obtained results of the experiment are not authoritative in the context of vibration tests—the element connected to the structure will behave differently than the element rigidly attached to the stand. However, they indicate that this type of research plays an important role in verifying Finite Element Method (FEM) model components based on measurements of fragmentary structures.

The authors refer to the dynamic analysis of thin-walled composite structures in [32], indicating numerous applications of these materials, including in the aviation industry. They indicate the need to determine the arrangement of reinforcement layers in the tested composites, which was confirmed by the obtained test results.

With regard to issues related to resonance frequencies, it was considered impossible to raise key messages related to their most important aspects. However, it has been done in the following section of this article. In this subsection, the natural frequency is estimated using the analytical approach by means of the Euler–Bernoulli theory.

The governing differential equation of motion was derived for the analytical estimation of the natural frequency by means of the Lagrange approach [33,34]:

(3) $$\frac{d}{dt}\left(\frac{\partial T}{\partial \dot{v}}\right)-\frac{\partial T}{\partial v}+\frac{\partial П}{\partial v}=0$$

where *T* and П are the kinetic and potential energies, respectively.

The geometrical relationships for the displacements, velocities, and curvature of the beam are expressed as a function of the deflection of the beam free end in form v(x,t)=ψ(x)v(t), where ψ(x) describes the beam deformation [35]:

(4) $$\psi \left(x\right)=\cosh\left(\lambda \frac{x}{L}\right)-\cos\left(\lambda \frac{x}{L}\right)+\eta \left(\sinh\left(\lambda \frac{x}{L}\right)-\sin\left(\lambda \frac{x}{L}\right)\right)\;\mathrm{and}\;\eta =-\frac{\sinh\lambda -\sin\lambda }{\cosh\lambda -\cos\lambda }$$

where the eigenvalue *λ* is obtained from the transcendental equation:

(5) 1+coshλcosλ=0

Introducing the corresponding derivatives of the kinetic *T* and potential *П* energies to the Lagrange (Equation (3)) and neglecting the third- and higher-order terms, the differential equation of motion is in form of [35]:

(6) $$\begin{array}{ll} {}[\varrho AN_{1}+\frac{h^{2}}{12}\rho AN_{4} & +\left(\rho AN_{3}+\frac{h^{2}}{12}\rho AN_{9}\right)v{\left(t\right)}^{2}]\ddot{v}\left(t\right)\\ & +\left(\rho AN_{3}+\frac{h^{2}}{12}\rho AN_{9}\right)\dot{v}{\left(t\right)}^{2}v\left(t\right)+EIN_{6}v\left(t\right)=-\varrho AN_{2}\ddot{q}\left(t\right) \end{array}$$

where constants *N_i_* in Equation (6) depend on the shape function and for the first mode shape are equal to:

(7) $$\begin{array}{cc} N_{1}=\overset{l}{\underset{0}{\int }}\psi {\left(x\right)}^{2}dx=0.25L\;\left[m\right], & N_{2}=\overset{l}{\underset{0}{\int }}\psi \left(x\right)ds=0.38L\;\left[m\right],\\ N_{3}=\int_{0}^{l}(\overset{s}{\underset{0}{\int }}{\left(\psi^{\prime }{\left(x\right)}^{2}dx\right)}^{2}dx=0.29/L\;\left[m^{-1}\right], & N_{4}=\overset{l}{\underset{0}{\int }}\psi^{\prime }{\left(x\right)}^{2}ds=1.16/L\;\left[m^{-1}\right],\\ N_{6}=\overset{l}{\underset{0}{\int }}\psi^{\prime \prime }{\left(x\right)}^{2}dx=3.09/L^{3}\;\left[m^{-3}\right], & N_{9}=\overset{l}{\underset{0}{\int }}\psi^{\prime }{\left(x\right)}^{4}ds=1.80/L^{3}\;\left[m^{-3}\right] \end{array}$$

To find the natural frequency of the beam, the linearized form of the equation of motion for free response was taken into account, as follows:

(8) $$\left[\varrho AN_{1}+\frac{h^{2}}{12}\rho AN_{4}\right]\ddot{v}\left(t\right)+EIN_{6}v\left(t\right)=0$$

Finally, the natural frequency for small vibrations from Equation (8) is given by the following:

(9) $$f_{n}=\frac{1}{2\pi }\sqrt{\frac{EIN_{6}}{\varrho A\left(N_{1}+\frac{h^{2}}{12}N_{4}\right)}}$$

Assuming that the analyzed composite structure has isotropic properties, one can apply its parameters (described in Table 1) for estimation of the natural frequency from Equation (9). In the case of the first mode shape, it is *f_n_* = 40 Hz.

### 2.4. The Test Stand for Dynamical Behavior

**Figure 4:**
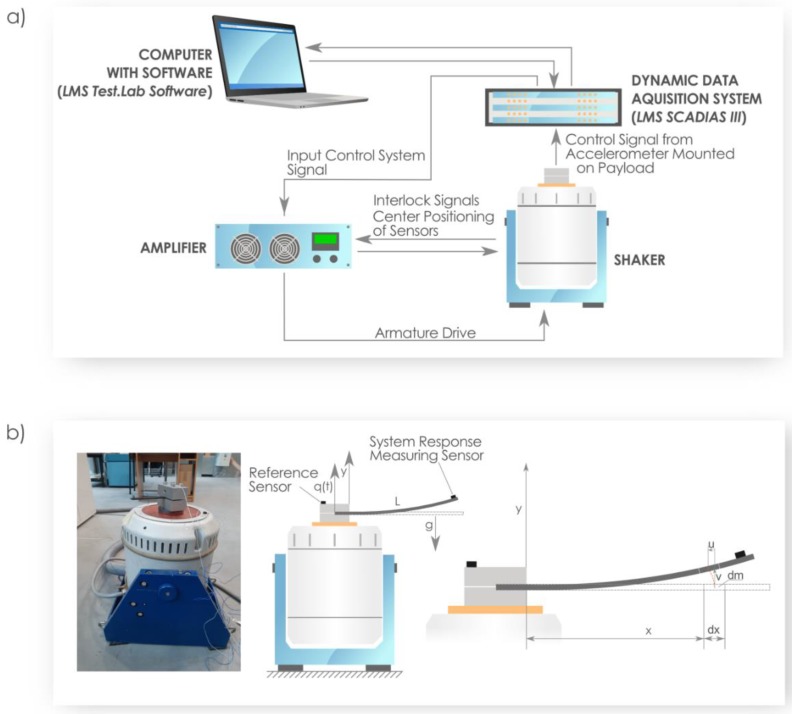
(**a**) The laboratory armature of the electro-dynamical vibration system. (**b**) The experimental set-up and the scheme of the vertically excited composite beam.

For the experiment performed in the laboratory, the electromagnetic shaker system, TIRAvib 50101, was used. A schematic of the measuring armature is presented in Figure 4a. The data acquisition system was based on the multichannel dynamic LMS Scadias III controller, which was supported by Test.Lab software. The armature reproduced environmental conditions in a range of specified frequency bands for the beam structure. The excitation input signal was assumed as a sinusoidal at constant level that corresponds to 1 *g*, where *g* corresponds to the gravity acceleration and is taken as *g* = 9.81 m/s^2^. The vibrations of the composite beam were measured in terms of the acceleration response by means of two acceleration sensors qualified for assumed frequency range (Figure 4b). The first sensor was installed on the shaker payload, which controlled the reference signal, while the second one located on the free end of the beam, and measured the on-time acceleration values of the composite structure [36].

## 3. Results

The conducted research allowed us to determine the properties of a polymer composite dedicated to aviation solutions in the field of issues related to tribology and in the scope of the base resonance zone. Detailed results are presented in the following two subsections.

### 3.1. Selected Tribological Properties

**Figure 5:**
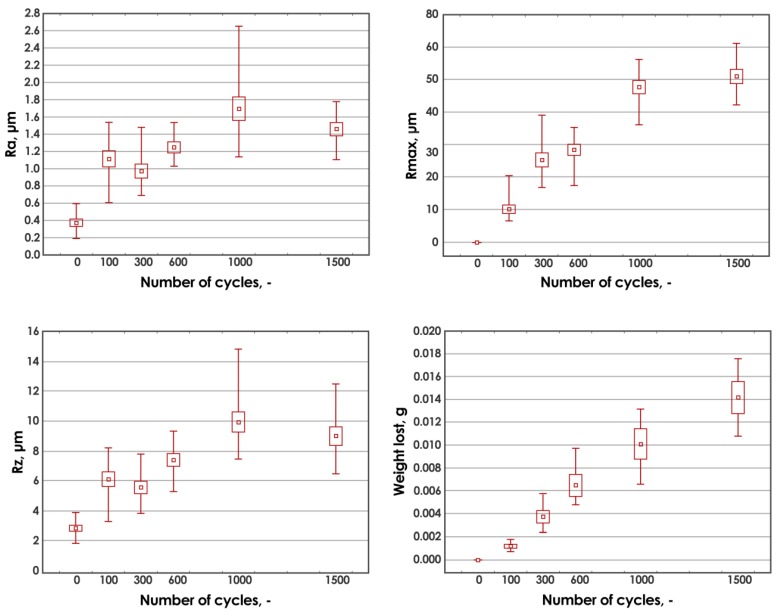
Dependencies of the arithmetical mean height (R_a_), the maximum height of the profile (R_z_), and maximum roughness depth (R_max_) parameters and weight loss on the number of wear cycles.

The results of the measurements of roughness parameters and abrasive wear are presented in Figure 5. As the number of cycles increases, the expected increase in abrasive wear (higher weight loss) and the largest depression R_max_ occur. The increase of mass loss has a linear character and is described by a regression function y = 9.3988 × 10^−6^ + 0.0005, with the coefficient of determination R^2^ = 0.9158. Similarly, there is a noticeable increase in the average roughness profile R_a_ and the largest R_z_ profile. However, changes in parameters R_a_, R_z_, and R_max_ are not linear. In up to 600 cycles, there is a slight stabilization of the values of these parameters, followed by their almost doubled increase and a slight decrease again. Noticeable fluctuations in roughness values are associated with the destruction of subsequent layers of carbon composite reinforcement. Larger changes in roughness are associated with the exposure of carbon fibers, which, in contact with the counter-sample, are damaged and crumble, causing more and more changes in the surface profiles.

The obtained tribological test results for the basic composite material constitute a starting point for further tests in the context of the dependencies between the modification of its composition and the observed abrasive wear. In addition, they are necessary to refer, in subsequent observations, to the impact of dependent variables (such as temperature, humidity, or UV radiation) on this material. The wide range of planned composition modifications and seasoning conditions that the composite does not release from highlight the need to conduct research on the basic material, thanks to which it will be possible to conduct final reliability and economic analyses.

### 3.2. Base Resonance Zone

**Figure 6:**
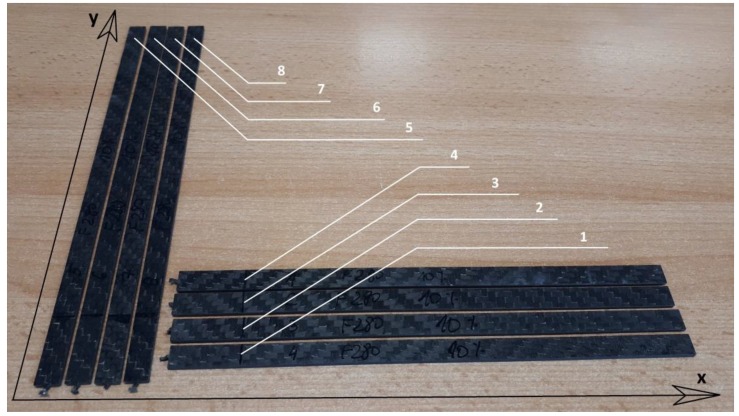
The sample cut along the x and perpendicular y directions.

The second part of the measurements concerns the dynamical analysis, which was performed in a small series of the polymer composites of four samples for two sets of fabric orientation. For the six layers of the fabric configuration 0/22.5/45/0/22.5/45 in the *x* direction, the composite plate was cut out in perpendicular directions to each other, as shown in Figure 6, where samples 1–4 are cut along the *x* direction and samples 5–8 along the *y* direction. Assuming the cutting directions at a right angle of the *x* and *y* axes, the dynamical behavior of the beam was investigated for both fabric configuration sets: 0/22.5/45/0/22.5/45 and 90/67.5/45/90/67.5/45. For such cut and prepared composite beams, experimental measurements were performed. The results of the amplitude–excitation frequency responses provide answer about the dynamical behavior of the samples, while the beam was vibrating in the vicinity of the first resonance frequency.

**Figure 7:**
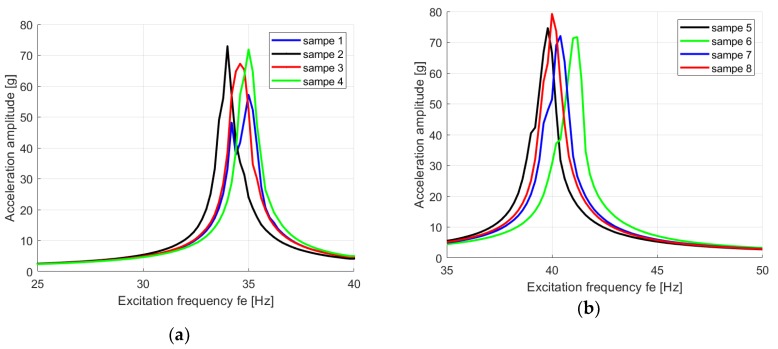
The response acceleration amplitude of the composite beams via excitation frequency at the first resonance zone of the samples cut along the x axis for the fabric configuration set 0/22.5/45/0/22.5/45 (**a**), and along the y axis for the fabric configuration set 90/67.5/45/90/67.5/45 (**b**).

Figure 7a,b presents the experimental results of the tests for both sets. Slight discrepancies are visible for each set of samples, which are caused by the beam thickness tolerance, as well as the clamping force on the shaker grip. It is clear that, for both sets of four samples, the cantilever beams reached the first mode shape at different excitation frequencies. The mean values of the resonance frequency for the first sample set (Figure 7a) is *f_e_* ≈ 34 Hz, but for the second set (Figure 7b) it equals *f_e_* ≈ 40 Hz, and this case is in accordance with the theoretically calculated excitation frequency from Equation (9). The dynamical analysis of the composite structure yielded a view about the stiffness of the material for one case of the fabric configuration, but was simultaneously excited in different (here, perpendicular) directions, as shown in Figure 6. The experiment revealed that the stiffness can be different. While the resonance frequency increased, the composite reached the progressive characteristic of the stiffness, and for decreasing resonance frequency, the material manifested the regressive characteristic. For the dimensions of the beams listed in Table 1, the measured response amplitudes of acceleration reached approximately *A_out_* ≈ 70 g at excitation force amplitude *A_in_* = 1 g. This shows that this polymer composite structure is able to carry significant dynamical loads, which is an advantage of this structure. One can conclude by linking these results to the real measurements on a PZL SW-4 helicopter, provided by the research in [37]. The authors recorded a frequency spectrum taken from the vertical stabilizer, while the helicopter was flying horizontally at a constant velocity of 200 km/h on 1000 m altitude. The measurements reported the range of frequencies and amplitudes to enable identifying the important design variables. From this investigation, one can select the sensitive frequencies and set these values against the results presented in Figure 7. While helicopter is flying, the noticeable vibration levels occurred at frequencies are 22, 30 and 45 Hz [37]. Analyzing the composite material of both fabric configuration set, the natural frequencies are close to those above. This is crucial from an assembly point of view in the manufacturing process. Because the fabric configuration set from the same composite material gives an opportunity to reach a different natural frequency, it moves the resonance point of the assembled parts applied in the aircraft construction. Namely, the parts assembled of the material from samples 1–4 are more convenient for use as elements which will be exposed to vibrations at about 45 Hz, but the other parts, including the configurations of samples 5–8 should be saved for use in the construction of parts exposed to excitations at 22 or 30 Hz.

## 4. Conclusions

The dynamical behavior of the polymer composite during vibrations in the vicinity of the base resonance zone reveals that the mechanical properties of the structure determined the considerable changes of the material amplitude–frequency response. This indicates the importance of fiber arrangement for applying the composites’ plate components in the aircraft structure. For the vibrating parts in particular, it should be verified that the natural frequency is safe for the sake of the excitation sources. It is noticeable, based on the comparison the vibration frequency spectra of the PZL SW-4 helicopter and the composite dynamical behavior reported in amplitude responses. The significant frequencies of the analyzed composite materials are close to each other. Consequently, the fiber arrangement is essential for changing the dynamical characteristics of the material, which allows us to move the resonance zone points for safe aircraft construction.

The increase in abrasive wear resulted in the expected increase in surface profile depression. At the maximum average weight loss at level 0.01416 g, the maximum of the largest recess in the material after the friction process R_max_ was 50.93 μm. No similar correlation was observed with the roughness coefficients—the arithmetical mean height (R_a_) and the maximum height of the profile (R_z_). These coefficients increased by leaps and bounds. Nevertheless, after 300 and 1500 cycles, decreased values were observed compared to the values at 100 and 1000 cycles. The decrease in the average R_a_ was 14.5% and 16.3%, respectively, for R_z_ (in both cases) it was 10%. The observed decrease indicates that temporary stabilization of the arithmetical mean height and maximum height of the profile was observed, which was adequate to the process of damage (breaking and falling out of carbon fiber and resin) of the subsequent carbon fabric layers in the composite.

Due to the exposition of the polymer composites used in aviation to harmful vibrations and the related friction process, it is necessary to conduct further research to improve the tribological properties by applying additional physical modifiers (e.g., Al_2_O_3_, SiC) while maintaining high resistance to damage caused by long-term dynamic loads.

This research confirmed the assumed hypothesis related to the abrasive wear process for the newly developed material, as well as empirically obtaining the dependencies of the resonance zone from the fabric orientation sets.

## Figures and Tables

**Table 1:** The parameters of the composite material beam.

Symbol and Value	Description
*ρ* = 2489 kg/m^3^	mass density of the beam
*E* = 42.40 GPa	Young modulus of the material
*L* = 200 mm	length of the beam
*b* = 10 mm	width of the beam
*h* = 2.4 mm	thickness of the beam
*A* = 24 mm^2^	cross section of the beam
*I* = 22.5 mm^4^	area moment of inertia
*N_1_* = 0.05 m	constant no1 depends on ψ(x)
*N_4_* = 5.80 m^−1^	constant no4 depends on ψ(x)
*N_6_* = 386.25 m^−3^	constant no6 depends on ψ(x)
